# The Efficiency of Hydroxychloroquine for the Treatment of Primary Sjögren’s Syndrome: A Systematic Review and Meta-Analysis

**DOI:** 10.3389/fphar.2021.693796

**Published:** 2021-09-07

**Authors:** Xuan Wang, Tongyangzi Zhang, Zizhen Guo, Jincheng Pu, Farooq Riaz, Run Feng, Xingxing Fang, Jiamin Song, Yuanyuan Liang, Zhenzhen Wu, Shengnan Pan, Jianping Tang

**Affiliations:** ^1^ Department of Rheumatology and Immunology, Tongji Hospital, Tongji University School of Medicine, Shanghai, China; ^2^ Department of Immunology, Shanghai Jiaotong University School of Medicine, Shanghai, China; ^3^ Department of Biochemistry and Molecular Biology, School of Basic Medical Sciences, Xi’an Jiaotong University Health Science Center, Xi’an, China

**Keywords:** hydroxychloroquine, Sjögren’s syndrome, systematic review, meta-analysis, treatmemt

## Abstract

**Objectives:** This meta-analysis was conducted to evaluate the effects of hydroxychloroquine (HCQ) in the treatment of primary Sjögren’s syndrome (pSS).

**Methods:** Nine databases were searched for data collection. We used clinical features, including involvement in superficial tissues and visceral systems, and experimental findings, including Schirmer’s test, unstimulated salivary flow rate (uSFR), C-reactive protein (CRP), erythrocyte sedimentation rate (ESR) and immunoglobulins (IgG, IgM and IgA) as major outcome measures. The Downs and Black quality assessment tool and RevMan 5.3 were used to assess the methodological quality and statistical analysis, respectively.

**Results:** Thirteen studies with pSS patients, consisting of two randomized controlled studies, four retrospective studies and seven prospective studies were analyzed. Results showed that HCQ treatment significantly improved the oral symptoms of pSS patients compared to non-HCQ treatment (*P* = 0.003). Similar trends favoring HCQ treatment were observed for uSFR (*p* = 0.05), CRP (*p* = 0.0008), ESR (*p* < 0.00001), IgM (*p* = 0.007) and IgA (*p* = 0.05). However, no significant improvement was observed in other clinical features, including ocular involvement, fatigue, articular lesions, pulmonary, neurological and lymphoproliferative symptoms, renal organs and other experimental parameters in the HCQ treatment group compared to the non-HCQ treatment group.

**Conclusion:** HCQ treatment showed moderate efficacy to improve oral symptoms, uSFR, ESR, CRP, IgM and IgA. However, HCQ could not alleviate organ-specific systemic involvement.

**Systematic Review Registration:**We have registered on the PROSPERO [https://www.crd.york.ac.uk/PROSPERO/], and the registration number is identifier [CRD42020205624]

## Introduction

Primary Sjögren’s Syndrome (pSS), with an estimated worldwide prevalence of 0.06% (Qin et al., 2015), is a chronic and systemic autoimmune disease that is characterized by focal lymphocytic infiltration of the exocrine glands causing oral and ocular dryness, fatigue and pain. These three symptoms are present in more than 80% of pSS patients and greatly compromise their quality of life (Meijer et al., 2009). In addition to the clinical manifestations of the salivary and lacrimal glands, a subset of patients also showed extra-glandular involvement with the development of signs and symptoms in other organs including skin, joints, lungs, gastrointestinal tract, kidneys, nervous and circulatory systems. These systemic complications occur in approximately 30–40% of pSS patients. Oral and ocular dryness is frequently assessed by measuring the unstimulated salivary flow rate (uSFR) and by Schirmer’s test. However, systemic complications often provide preliminary clues to diagnose pSS. Moreover, anti-SSA/Ro (Sjögren’s syndromeA) antibodies, often associated with anti-SSB/La (Sjögren’s syndromeB) antibodies, should be assessed in suspected patients. However, in the absence of anti-SSA antibodies, biopsy of minor salivary glands is typically recommended to establish a diagnosis of pSS. Furthermore, laboratory indices including erythrocyte sedimentation rate (ESR), C-reactive protein (CRP) and detection of immunoglobulins (IgG, IgM and IgA) are often used as markers to indicate disease development and activity (Shiboski et al., 2017).

Currently, there is no drug that can cure pSS. Treatment goals are symptom palliation and prevention of complications. Thus, it is necessary for rheumatologists to design and identify potential immunosuppressive therapeutic drugs to treat pSS (Mariette and Criswell, 2018). Currently, the therapeutic agents that are commonly used to treat pSS are hydroxychloroquine (HCQ), prednisone, methotrexate, mycophenolate sodium, azathioprine and cyclosporine. The emergence of biological agents has raised expectations for a therapeutic response in pSS. However, their usage is limited due to the lack of licensing and safety evidence. Among the available therapeutic options, HCQ has a good safety profile with minimal side effects (Brito-Zerón et al., 2019).

HCQ, an approved drug to prevent or cure malaria, is now considered a common immunomodulatory drug which is widely used for treating systemic lupus erythematosus (SLE) and rheumatoid arthritis (RA) (Suarez-Almazor et al., 2000; Ruiz-Irastorza et al., 2010). Recent research illustrated possible mechanisms (Schrezenmeier and Dörner, 2020). At the pharmacological level, HCQ has the ability to accumulate in acidic compartments such as lysosomes, as well as inflamed (acidic) tissues. At the cellular level, inhibition of autophagy prevents immune activation of different cell types, which inhibits cytokine production and modulates CD154 expression on T cells may be its important mechanism. Some studies have shown that HCQ is effective in the treatment of pSS (Fox et al., 1996). Cumulative evidence has suggested that HCQ significantly improves the ocular symptoms, particularly laboratory indications (Tishler et al., 1999; Rihl et al., 2009; Çankaya et al., 2010; Yavuz et al., 2011) and protects against systematic damage (Demarchi et al., 2017; Hernández-Molina et al., 2018). However, a randomized trial of HCQ, as compared with placebo, showed no significant improvement in the symptoms of pSS during 24 weeks of treatment (Gottenberg et al., 2014). Similarly, several other studies also concluded that HCQ is not an effective treatment for pSS (Kruize et al., 1993; Yoon et al., 2016). A recent published meta-analysis stated that there was no significant difference observed between placebo and HCQ-treated groups for the treatment of dry mouth and dry eyes in pSS (Wang et al., 2017). In contrast, HCQ is frequently used in China for the treatment of pSS (according to a multi-center registration study, about 67.5% of pSS patients used this drug) (Xu et al., 2020); however, there is no consensus of its efficacy. Given the contradictory conclusions for the use of HCQ, we intended to preliminarily evaluate the value of HCQ in the treatment of pSS through a literature review.

## Methods

### Procedures

This review was performed following the preferred reporting items for systematic reviews and meta-analyses (PRISMA) guidelines (Moher et al., 2015). Meanwhile, we have registered on the PROSPERO and the registration number is CRD42020205624.

### Literature Search

**FIGURE 1:**
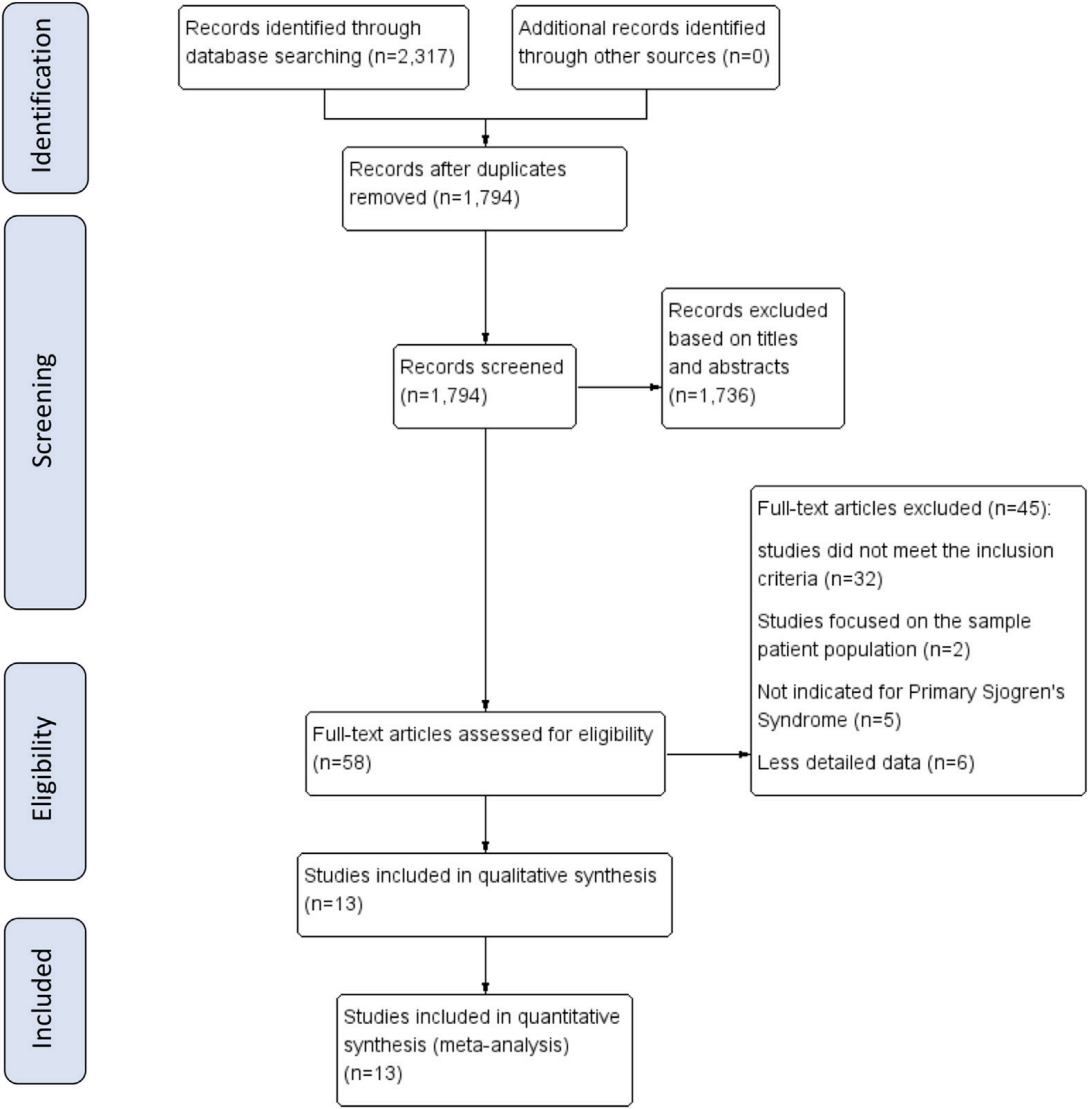
Flow chart of inclusion and exclusion criteria and study selection.

Two authors independently searched Medline, Embase, Cochrane Central Register of Controlled Trials, LILACS (Latin American and Caribbean Health Sciences Literature), Wanfang Med Database, China National Knowledge Infrastructure, Chinese VIP Information Database and Chinese Biomedical. Meanwhile, we searched related studies in Clinicaltrials to confirm the availability of relevant unpublished studies. The languages were restricted to English and Chinese. For English databases, subject headings and text-word searches were used, and the search details included “sjogren syndrome,” “sjogren’s syndrome,” “sjogrens syndrome,” “sicca syndrome,” “sjogren’s,” “ss,” “hydroxychloroquine,” “chloroquine,” “HCQ,” “antimalarial,” “plaquenil,” “treatment,” “therapy,” “therapeutics,” “management” and “treat”. For Chinese databases, free text terms were used, such as “gan zao zong he zheng” (which means Sjögren’s syndrome in Chinese) and “qiang lv kui” (which means hydroxychloroquine in Chinese). Studies included in this paper were published before September 30, 2020 and were confined to humans. The search strategies are detailed in Figure 1.

### Inclusion Criteria

1) Types of studies: All randomized controlled clinical trials (RCTs) or observational studies investigating the use of HCQ for pSS were included, regardless of blinding, types or languages of publication. 2) Types of participants: Patients regardless of sex, age or ethnicity, who met the diagnostic criteria according to the international classification of pSS in 2002/2012/2016 (Downs and Black, 1998; Vitali et al., 2002; Shiboski et al., 2012). 3) Types of interventions: In the control study, the experimental group was treated with HCQ monotherapy and the control group was not treated with HCQ or any other immunosuppressants (placebos-control or self-control). In observational studies, baseline data before the administration of HCQ were recorded as the non-HCQ group. 4) Types of outcome measures: The number of patients with clinical symptoms involving the oral domain, ocular domain, fatigue, articular domain, pulmonary domain, neurological domain, lymphoproliferative domain and renal domain, which were binomial variables. Objective indicators included Schirmer’s test, uSFR, CRP, ESR, and levels of immunoglobulin (IgG, IgM and IgA), which were continuous variables. Whether those domains were involved were all determined according with the international classification of pSS in 2002/2012/2016 (Downs and Black, 1998; Vitali et al., 2002; Shiboski et al., 2012).

### Exclusion Criteria

1) Incomplete data, or data impossible to extract (If the number of patients or the effective rate of HCQ treatment for subjective symptoms was not provided or not available according to original data, the research would be excluded. Research regarding to objective indices without control group data. would be excluded from the analysis); 2) Various studies focused on the same patient sample population or which used the same data repeatedly; 3) Nonhuman studies; 4) Reviews, case reports, comments, meeting minutes.

### Data Extraction and Quality Assessment

The literature was exported to Endnote X8 excluding any repeated literature. Later, two authors (Xuan Wang and Tongyangzi Zhang) independently screened the remaining literature. Initial screening was conducted by reviewing the titles and abstracts and was further confirmed by reading the full text which was then cross-checked. Any discrepancy was resolved by discussion with the third author (Zizhen Guo). The full texts of potential studies were accessed to decide if those should also be included.

Two authors extracted the data from the studies. Briefly, all relevant data from individual studies including author, year of publication, study parameters, characteristics of patients, detailed interventions, outcomes and adverse effects were extracted and summarized in tabular form.

The quality of the studies was assessed using the Downs and Black quality assessment tool that contains a list of 27 questions for evaluation of the reporting, external validity, internal validity-bias, confounding (selection bias) and the power of assessed studies (Downs and Black, 1998). The level of evidence represented by each study was categorized based on the Oxford Centre for Evidence-Based Medicine Levels of Evidence (OCEBM; http://www.cebm.net/index.aspx?o=5653). The OCEBM classifies the evidence levels of the research into five grades, ranging from level 1 to level 5.

### Statistical Analysis

All extracted data were entered into Review Manager 5.3 software and subjected to a meta-analysis. For dichotomous variables, individual and pooled statistics were calculated as Odds Ratio (OR) with 95% confidence intervals (CI). For continuous outcomes, individual and pooled statistics were calculated as mean differences (MD) or standard mean differences, as indicated, with 95% CI. Heterogeneity was evaluated with the Homogeneity Test (Q test; α = 0.1) and quantified with *I* ^*2*^. The fixed effects model was used when *p* ≥ 0.10 and *I* ^*2*^ ≤ 50%, which suggested the homogeneity was appropriate for meta-analysis. Otherwise, a random effects model was used.

### Assessment of Clinical Heterogeneity and Sensitivity Analysis

The trial characteristics that could influence the effect of the observed treatment were examined. Clinical heterogeneity was investigated using a sensitivity analysis. After removing the studies with the lowest quality, the combined value was re-calculated for the analysis of sensitivity.

## Results

### Characteristics of Included Studies

Electronic searches retrieved a total of 2,317 citations. We removed 523 duplicated articles and excluded 1,781 articles after screening the abstracts. Finally, 13 studies (Yavuz et al., 2011; Çankaya et al., 2010; Rihl et al., 2009; Tishler et al., 1999; Demarchi et al., 2017; Hernández-Molina et al., 2018; Gottenberg et al., 2014; Kruize et al., 1993; Yoon et al., 2016; Li et al., 2013; Shuai and Sun, 2011; Dong, 2012; Shi et al., 2008) (nine studies published in English, four studies published in Chinese, in Table 1) were included in this meta-analysis, which included 987 pSS patients (951 were female). Eight (Tishler et al., 1999; Shi et al., 2008; Rihl et al., 2009; Çankaya et al., 2010; Shuai and Sun, 2011; Yavuz et al., 2011; Dong, 2012; Li et al., 2013) of the 13 studies did not use a control group, thus we extracted the data for the baseline from the non-HCQ group data. The sample size ranged from 14 to 377 patients in each study, whereas the treatment duration ranged from 12 weeks to 6 years. The characteristics of the selected studies are shown in Table 1.

**TABLE 1:** Characteristics of included studies.

Author	Year	Region	Study design	Age (year)	Gender (M/F)	Dosage of HCQ	Treatment duration	Main outcome
Kruize AA, et al.	1993	Netherlands	Prospective	51.9 ± 15.5	0/19	400 mg/d	12 m	Oral damage, ocular involvement, fatigue, articular lesions, Schirmer’s test, ESR, IgG, IgM, IgA
Gottenberg JE, et al.	2014	France	RCT	48.9 ± 12.7	10/110	400 mg/d	24 w	Oral damage, ocular involvement, fatigue, articular lesions, pulmonary, neurological, lymphoproliferative, renal organs, Schirmer’s test, uSFR, CRP, ESR, IgG, IgM, IgA
Yoon CH, et al.	2016	Korea	RCT	56.8 ± 9.66	0/26	300 mg/d	16 w	Schirmer’s test, ESR
Demarchi J, et al.	2017	Argentina	Retrospective	55.7 ± 14	6/215	400 mg/d	12 m	Fatigue, articular lesions, pulmonary, neurological, lymphoproliferative, renal organs
Hernández-Molina G, et al.	2018	Latin-American Argentina (n = 110), Brazil (n = 49), Mexico (n = 218)	Retrospective	48.9 ± 12.7	10/367	400 mg/d	6 y	Oral damage, ocular involvement, pulmonary, neurological, lymphoproliferative, renal organs
Yavuz S, et al.	2011	Turkey	Prospective	56 ± 14	0/32	6.5 mg/kg	12 w	Schirmer’s test
Cankaya H, et al.	2010	Turkey	Prospective	48.9 ± 10.5	0/30	400 mg/d	18 w	Oral damage, uSFR
Rihl M, et al.	2009	Germany	Retrospective	56 ± 12.4	0/14	300 mg/d (50–64 kg)	4.9 ± 1.1 m	Schirmer’s test, IgG, IgA
						400 mg/d (>64 kg)		
Tishler M, et al.	1999	Israel	Retrospective	58 ± 10.3	0/14	200 mg/d	12 m	CRP, ESR, IgG, IgM, IgA
Li SK, et al.	2013	China	Retrospective	43.27 ± 6.18	3/26	400 mg/d	>6 m	CRP, ESR, IgG, IgM
Shuai SQ, et al.	2011	China	Retrospective	45.38 ± 17.21	2/28	400 mg/d	>6 m	CRP, ESR, IgG, IgM
Dong SQ, et al.	2012	China	Retrospective	45.11 ± 9.57	4/31	400 mg/d	>6 m	CRP, ESR, IgG, IgM
Shi Q, et al.	2008	China	Retrospective	21–65	1/39	400 mg/d	>6 m	ESR, IgG, IgM, IgA

M, male; F, female; RCT, randomized clinical trial; HCQ, hydroxychloroquine; ESR, erythrocyte sedimentation rate; immunoglobulin (IgG, IgM, and IgA); uSFR, unstimulated salivary flow rate; CRP, C-reactive protein.

### Quality Assessment of Included Studies

Based on the Downs and Black criteria, the median methodological quality score for all thirteen studies was 25/32 (range = 21–31). None of these studies achieved a full score. However, based on OCEBM levels of evidence, two studies (Gottenberg et al., 2014; Yoon et al., 2016) belonged to Level 2, six studies (Kruize et al., 1993; Shi et al., 2008; Shuai and Sun, 2011; Dong, 2012; Li et al., 2013; Demarchi et al., 2017; Hernández-Molina et al., 2018) belonged to Level 3, and three studies (Tishler et al., 1999; Rihl et al., 2009; Çankaya et al., 2010; Yavuz et al., 2011) belonged to Level 4 (Table 2).

**TABLE 2:** Quality assessment of included studies.

Author	Year	Study design	Downs and black quality score						OCEBM levels of evidence
			Reporting	External validity	Internal validity - bias	Internal validity-confounding	Power	Total	
			11	3	7	6	5	32	
Kruize AA, et al.	1993	prospective	11	3	7	4	3	28	3
Gottenberg JE, et al.	2014	RCT	10	3	7	6	5	31	2
Yoon CH, et al.	2016	RCT	11	3	7	6	4	31	2
Demarchi J, et al.	2017	retrospective	11	3	5	6	5	30	3
Hernández-Molina G, et al.	2018	retrospective	9	3	5	3	5	25	3
Yavuz S, et al.	2011	prospective	9	3	5	2	4	23	4
Cankaya H, et al.	2010	prospective	9	3	5	3	4	24	4
Rihl M, et al.	2009	retrospective	10	3	5	4	3	25	4
Tishler M, et al.	1999	retrospective	9	2	5	2	3	21	4
Li SK, et al.	2013	Retrospective	9	2	5	3	3	22	3
Shuai SQ, et al.	2011	Retrospective	10	2	5	2	3	22	3
Dong SQ, et al.	2012	Retrospective	10	2	5	3	3	23	3
Shi Q, et al.	2008	Retrospective	9	2	5	3	3	22	3

RCT, randomized controlled clinical trial; OCEBM, Evidence-Based Medicine Levels of Evidence.

### Effects of Interventions

#### The Effect of HCQ on Clinical Symptoms

**FIGURE 2:**
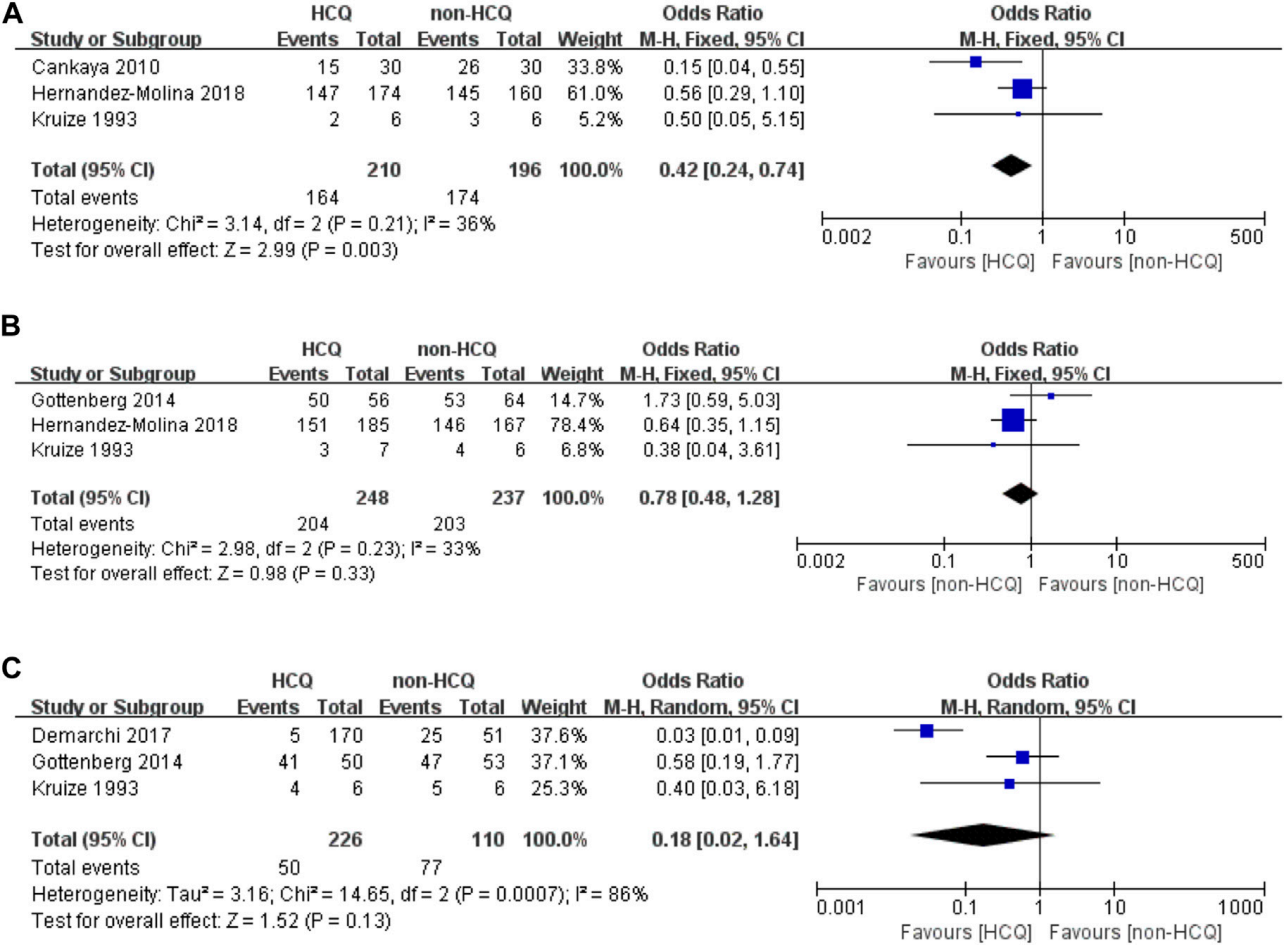
Forest plot of studies comparing HCQ group and the non-HCQ group, examining the effect on pSS (including oral domain, ocular domain and fatigue). **(A)** oral domain. **(B)** ocular domain. **(C)** fatigue.

As shown in Figure 2, three studies reported changes in symptoms of the oral domain with an acceptable heterogeneity (*p* = 0.21, *I* ^*2*^ = 36%). This indicated that HCQ treatment can significantly improve the symptoms of the oral domain in pSS compared to non-HCQ treatment (OR 0.42, 95% CI 0.24–0.74, *p* = 0.003). Moreover, three studies reported changes in symptoms of the ocular domain with an acceptable heterogeneity (*p* = 0.23, *I* ^*2*^ = 33%). These results revealed no significant difference between the two groups (OR 0.78, 95% CI 0.48–1.28, *p* = 0.33). Similarly, changes in fatigue were reported in three studies, with significant heterogeneity (*p* = 0.0007, *I* ^*2*^ = 86%). These results revealed that there was no obvious difference between the two groups (OR 0.18, 95% CI 0.02–1.64, *p* = 0.13).

**FIGURE 3:**
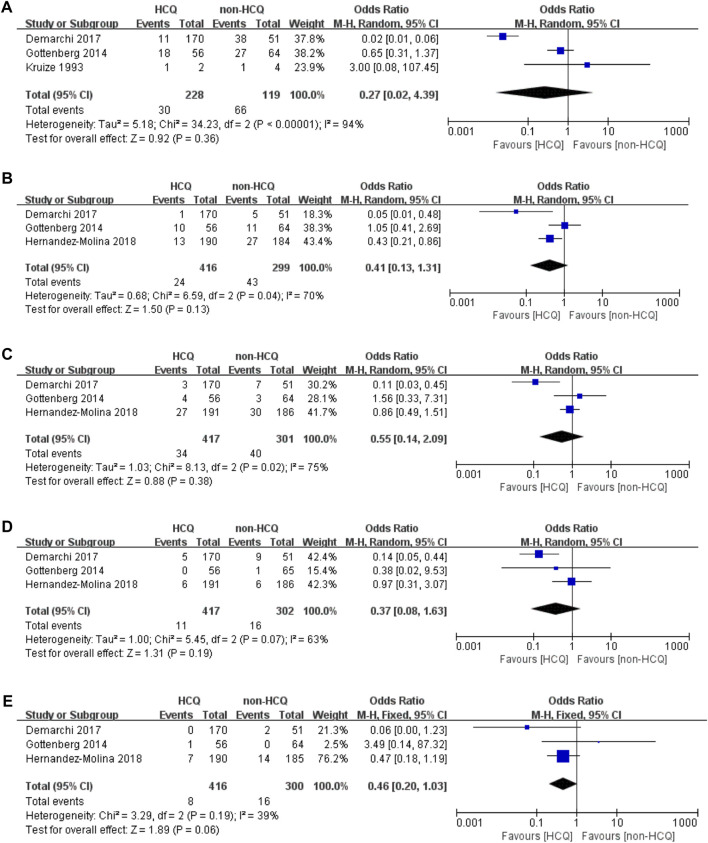
Forest plot of studies comparing HCQ group and the non-HCQ group, examining the effect on pSS (including articular domain, pulmonary domain, neurological domain, lymphoproliferative domain and renal domain). **(A)** articular domain. **(B)** pulmonary domain. **(C)** neurological domain. **(D)** lymphoproliferative domain. **(E)** renal domain.

We further analyzed organ-specific systemic involvements (Figure 3). The articular domain involvements, mentioned in three studies, had significant heterogeneity (*p* < 0.00001, *I* ^*2*^ = 94%). These results revealed no obvious difference between the two groups (OR 0.27, 95% CI 0.02–4.39, *p* = 0.36). For pulmonary domain involvements, mentioned in three studies, there was significant heterogeneity (*p* = 0.04 *I* ^*2*^ = 70%). These results revealed no obvious difference between the two groups (OR 0.41, 95% CI 0.13–1.31, *p* = 0.13). For neurological domain involvements, mentioned in three studies, there was significant heterogeneity (*p* = 0.02, *I* ^*2*^ = 75%). These results revealed no obvious difference between the two groups (OR 0.55, 95% CI 0.14–2.091, *p* = 0.38). The lymphoproliferative domain involvements, mentioned in three studies, had significant heterogeneity (*p* = 0.07, *I* ^*2*^ = 63%). These results revealed no obvious difference between the two groups (OR 0.37, 95% CI 0.08–1.63, *p* = 0.19). Finally, we analyzed that the renal domain involvements, mentioned in three studies, which had acceptable heterogeneity (*p* = 0.19, *I* ^*2*^ = 39%). Similar to the previous findings, these results also revealed that there was no obvious difference between the two groups (OR 0.46, 95% CI 0.20–1.03, *p* = 0.06).

#### The Effects of HCQ on Objective Indicators

**FIGURE 4:**
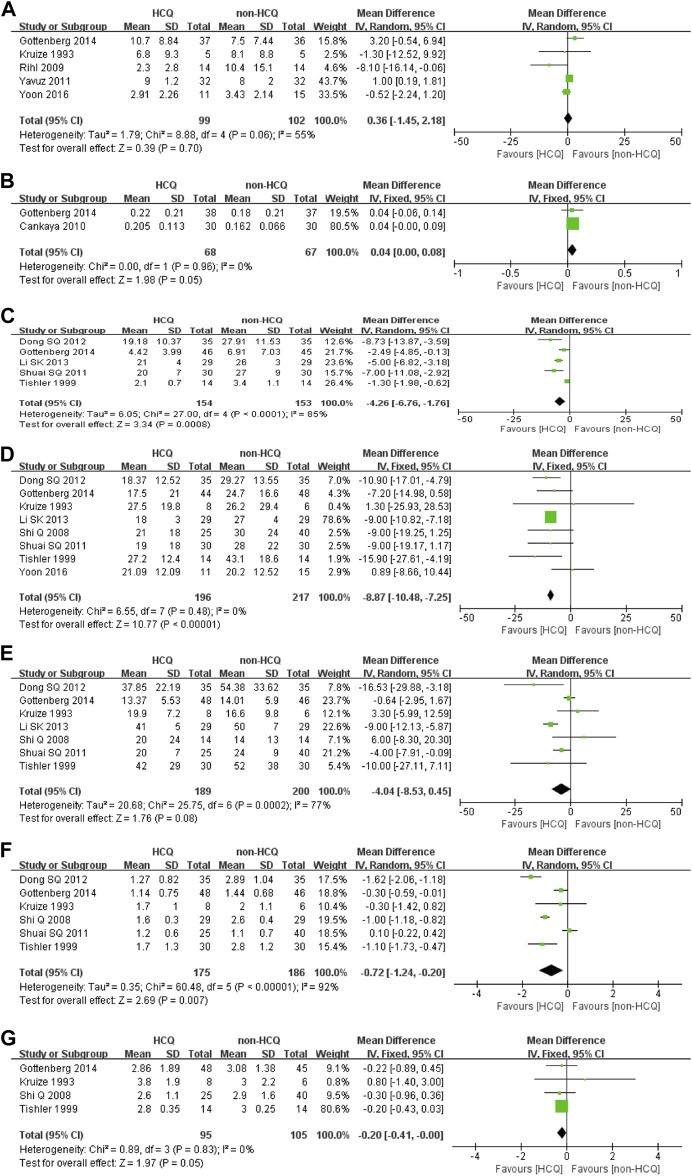
Forest plot of studies comparing HCQ group and the non-HCQ group, examining the effect on pSS (including Schirmer’s test, uSFR, CRP, ESR, IgG, IgM and IgA). **(A)** Schirmer’s test. **(B)** uSFR. **(C)** CRP. **(D)** ESR. **(E)** IgG. **(F)** IgM. **(G)** IgA.

As illustrated in Figure 3 and Figure 4, we further analyzed objective indicators of pSS. Two studies recorded data of the uSFR and five studies recorded CRP data, and pooled results showed that after HCQ treatment, uSFR values increased significantly (MD 0.04, 95% CI 0.00–0.08, *p* = 0.05) and CRP declined significantly (MD −4.26, 95% CI −6.76–−1.76, *p* = 0.0008). Moreover, eight studies reported changes in ESR. It was found that ESR was significantly reduced after HCQ treatment (MD −8.87, 95% CI −10.48–−7.25, *p* < 0.00001) with low heterogeneity (*p* = 0.48, *I* ^*2*^ = 0%). The same trend favoring HCQ treatment was found for IgM (MD −0.72, 95% CI −1.24–−0.20, *p* = 0.007) and IgA (MD −0.2, 95% CI −0.41–0.00, *p* = 0.05). However, there was no statistically significant difference in Schirmer’s test (MD −0.36, 95% CI −1.45–2.18, *p* = 0.7) or in IgG (MD −4.04, 95% CI −8.53–0.45, *p* = 0.08).

#### Sensitivity Analysis and Publication Bias

The studies analyzed in this meta-analysis were less than 10, therefore, we did not undertake visual inspection of funnel plots as indicators of publication bias (Higgins and Wells, 2011). However, we performed sensitivity analysis by removing the low quality studies and then compared the differences between the results. These results showed that there was no effect on the outcome.

## Discussion

This meta-analysis was conducted based on thirteen studies containing 987 pSS patients. Our results showed significant therapeutic effects of HCQ in pSS which improved the oral domain symptoms as well as several objective indicators such as uSFR, ESR, CRP, IgM and IgA. However, the efficacy of HCQ in pSS was not evident regarding ocular domain symptoms, fatigue, Schirmer’s test and IgG. Moreover, HCQ did not improve extra-glandular involvements in the articular, pulmonary, neurological, lymphoproliferative or renal domains.

Regarding the oral domain symptoms in pSS, three of the included studies described dry mouth and buccal mucosa dryness as general indicators of the oral domain (Kruize et al., 1993; Çankaya et al., 2010; Hernández-Molina et al., 2018). Our combined statistics suggested that HCQ treatment was effective in improving oral symptoms, but no similar results have been reported in other studies, to the best of our knowledge. Simultaneously, our results showed that uSFR was improved after treatment with HCQ, which was consistent with the improvement in oral symptoms. In 2011, Yavuz et al. (2011) demonstrated that HCQ may alleviate the symptoms of dry eyes in pSS, but in 2016, Yoon et al. (2016) found no significant differences in dry eyes after HCQ treatment. Both studies, however, were based on ocular examinations such as Schirmer’s test and average tear drop, with no definition of the primary outcome for ocular symptoms. Although three studies (Shi et al., 2008; Shuai and Sun, 2011; Li et al., 2013) from China mentioned that HCQ improved patients’ clinical symptoms such as dry eyes, dry mouth and joint pain, we were unable to extract data for statistical analysis due to their use of visual analogue scale scores rather than the number of patients with mitigation of clinical symptom such as dry eyes and mouth.

Among the studies included in our research, three studies analyzed ocular domain symptoms (Kruize et al., 1993; Gottenberg et al., 2014; Hernández-Molina et al., 2018) and five studies reported changes in Schirmer’s test (Kruize et al., 1993; Rihl et al., 2009; Yavuz et al., 2011; Gottenberg et al., 2014; Yoon et al., 2016). However, these studies indicated that treatment with HCQ was ineffective. Most of the selected studies analyzed markers including ESR, CRP and immunoglobulin (IgG, IgA, IgM) to indicate disease activity. Our research found that ESR, CRP, IgA and IgM were significantly improved after HCQ treatment, which represents the immune modulating functions of HCQ for pSS. In addition, we found that only four (Kruize et al., 1993; Gottenberg et al., 2014; Demarchi et al., 2017; Hernández-Molina et al., 2018) of the included studies related to extra-glandular manifestations. Two (Gottenberg et al., 2014; Hernández-Molina et al., 2018) of these used the EULAR Sjögren’s Syndrome Disease Activity Index, a validated measure to assess the activity and outcome of pSS, to assess the systemic complications of the disease (Seror et al., 2011). Thus, even though extra-glandular manifestations are considered important changes and indicators in pSS, only a few studies have investigated these indicators.

We further observed that all thirteen studies included in our study had several deficiencies. First, the design and criteria for assessment of these studies were very different. Only two of them were RCTs (Gottenberg et al., 2014; Yoon et al., 2016), while seven studies were prospective trials (Kruize et al., 1993; Shi et al., 2008; Çankaya et al., 2010; Shuai and Sun, 2011; Yavuz et al., 2011; Dong, 2012; Li et al., 2013) and four were retrospective trials (Tishler et al., 1999; Rihl et al., 2009; Demarchi et al., 2017; Hernández-Molina et al., 2018). Most of these studies did not describe the specific randomization and the detailed allocation concealment and blinding schemes. Some studies only described changes in subjective symptoms, while others only analyzed data representing changes in objective indicators. Although all the studies included in the analysis were met the diagnostic criteria, most of them adopted self-designed efficacy standards to evaluate the effects, especially different definitions to describe clinical symptoms. Meanwhile, some indicators could not be correlated statistically. Thus, we could not fully analyze the accuracy of their results. Second, differences in the dosage of HCQ and trial duration may have affected the results. The dosage of HCQ in the included studies ranged from 200 to 400 mg/day, and the study durations ranged from 12 weeks to 6 years. This may have increased the heterogeneity in statistical analysis. Third, most trials were conducted on a limited number of patients. Only three studies consisted of more than 100 patients (Gottenberg et al., 2014; Demarchi et al., 2017; Hernández-Molina et al., 2018), while most of the studies were limited to less than 30 participants, and all four Chinese studies we included small sample sizes.

Several limitations of our study should also be noted. We included Chinese studies, none of them had extractable data on clinical manifestations, which may have caused bias in the combined statistics. On the other hand, given the small number of RCTs, we decided to include observational studies. Because of the lack of a control group, the baseline data from these studies were compared with the data after HCQ administration, which may have resulted in considerable variation in the quality of articles and increased the possibility of heterogeneity. In summary, our study provided partial evidence supporting the use of HCQ in pSS and suggesting that uniform and well-designed standard trials are needed to verify these therapeutic effects.

## Conclusion

In this meta-analysis, we evaluated the efficacy of HCQ treatment in pSS. HCQ treatment showed significant efficacy in improving oral symptoms, uSFR, as well as inflammatory indices (ESR, CRP) and immunoglobulins (IgM). However, the use of HCQ did not improve the organ-specific systemic involvements in the articular, pulmonary, neurological, lymphoproliferative and renal domains. Based on current research progress, only limited clinical trials have been conducted to evaluate the efficacy of HCQ in pSS. Therefore, given the wide use of HCQ in clinics to treat pSS, well-designed randomized and controlled trials will be required to provide higher quality evidence to confirm these findings.

## Data Availability

The original contributions presented in the study are included in the article/Supplementary Material, further inquiries can be directed to the corresponding author.
